# Comparison of the Circular and Flap Operations in Amputating the Thigh

**Published:** 1834-07-01

**Authors:** Herbert Mayo

**Affiliations:** Surgeon to the Middlesex Hospital.


*Comparison of the Circular and Flap Operations in amputating the Thigh.* From *Clinical Remarks,* by HERBERT MAYO, F.R.S., Surgeon to the Middlesex Hospital.

THE occurrence, in the Middlesex Hospital, during the last half year, of several cases requiring amputation of the thigh, has offered our students, along with other points of interest, an opportunity of comparing the advantages of the circular and flap operations.

I. After amputation of the thigh, the surgeon is often disappointed at finding that, careful as he may have been to guard against this particular evil, *the bone projects*, and, either becoming uncovered, throws off a ring of exfoliation, or, being just retained within the integuments, after healing, rests immediately against the cicatrix; the stump is conical, and unable to sustain pressure.

The following case will serve to exemplify what I have stated.

— Devonport, ætat. twenty-five, underwent amputation of the thigh in December last, for disease of the knee. The operation performed was the double circular incision. The integuments and fascia lata were divided by one incision. After these parts had been turned back, the muscles were divided by a second incision. The flesh was then pushed back from the bone, and the bone was sawn through. The edges of the integument, when drawn together, met well in a transverse line. Inflammation of the face of the stump, however, supervened on the third day; when it became necessary to remove the roller and adhesive plaster. No union by adhesion took place; the whole cavity of the stump suppurated; the bone projected; and it was evident that the sawn edge, for the depth of a quarter of an inch, would exfoliate. While the inflammation continued, little could be done to prevent the increasing retraction of the integument and muscles; but, when it had subsided, means were used, by rollers laid longitudinally on each side, and fixed by a circular roller round the thigh, to get some purchase upon the integuments and muscles. The longitudinal rollers were fixed to the amputation-cradle in a manner to exert a constant traction upon the flesh of the thigh, which they gradually brought forward, so that, at the expiration of ten weeks, when the rim of bone had exfoliated, the end of the stump was tolerably full and round.

In this case, inflammation casually supervening on the third day appeared the source of all the mischief: but it is possible that it had an earlier origin; and I am not satisfied that, in the first dressing, the roller was not applied too tightly round the thigh, in the wish of keeping the muscles and integuments well forward over the bone, which, though covered, yet seemed disposed to project. The bone may have been left of inconvenient length, and the attempt by pressure to support the integuments covering it may have promoted the inflammation which followed.

The patient's recovery was protracted and tedious; but was this wholly, or exclusively, owing to the too great length and subsequent exfoliation of the bone?

Jane Deans, ætat. seventeen, suffered amputation of the thigh on the 7th of February. The steps of the operation were the same as in the preceding case; but, on preparing to close the stump, on looking closely to the length of the bone, the same tendency to protrude was apparent as in the preceding instance. I therefore sawed off an additional third of an inch of bone. The sides of the stump then came together excellently, the end of the bone being completely buried in muscle, and the integuments meeting without the least strain over all. The stump was then dressed very lightly.

I was extremely disappointed with the immediate progress of this case: the patient, at the time of the amputation, was in a state apparently the most favourable for union by adhesion. Originally a very healthy young woman, of rather a full habit, she had suffered for many months severe pain, which had reduced, but had not dangerously extenuated her. The loss of blood in the operation, and removal of the source of pain, should have tended to produce that repose in her system, which would have encouraged the speediest healing of the stump. The reverse happened: the whole interior of the stump suppurated, and, although the patient's health was excellent after the first few days, yet nothing could be more tedious than the union of the wound: the final closing of the stump did not take place for twelve weeks. Nevertheless, as it drew together, the extremity was full, round, and fleshy; the cicatrix a mere line, two inches in length.

Two conclusions the student might deduce from the preceding cases: one, that the stump produced after amputation of the thigh by the circular incisions may have the greatest possible roundness or fulness; the other, that a surgeon, if he has a doubt as to the length of the bone, when he is about to close the stump, should not hesitate to saw off an additional third or half inch. It is needless to say, that the sawing off a second portion of bone is no sensible aggravation of the patient's sufferings. The only objection to it, and it is of trifling moment, is that the second section may be followed by bleeding from the medullary vessels, which may render an additional delay of some minutes necessary before the stump can be closed.

But *ought* there ever to be a necessity for sawing off a second portion of bone? In some instances I believe it to be unavoidable, supposing the surgeon to operate with a tourniquet. Without a tourniquet, indeed, the full retraction of the muscles takes place immediately, and the surgeon sees exactly at what height it is requisite to saw the bone. But, in operations with the tourniquet, this point cannot in every instance be determined. I have tried the effect of waiting two or three minutes after the division of the soft parts, the tourniquet remaining on, before I sawed through the bone; but the result disappointed me. I sawed through the bone, after having so waited, at what appeared to me a proper length; but, upon removing the tourniquet, additional retraction took place, and the bone was too long by a third of an inch.

In studying to obtain a sufficiently full and fleshy stump by the circular operation, a choice of evils has therefore to be made: it is necessary either to operate without a tourniquet, or occasionally to saw off an additional portion of bone. In some cases, perhaps, it may be well to prefer amputation without the tourniquet. If you have an assistant on whom you can perfectly depend, and if it be not important to secure the patient against unnecessary loss of blood, the operation without a tourniquet is certainly more rapid, and *then* almost as safe: otherwise, and for general practice, the operation with the tourniquet ought alone to be recommended. There is, indeed, a third method, which, however, would be attended with its own sources of inconvenience,—to divide the soft parts,—to tie the vessels,—then to remove the tourniquet,—and, as the last step, to saw the bone.

II. My disappointment at the tedious recovery of the last patient led me to employ the flap operation at the next opportunity. I thought it possible that, in the operation by circular incisions, the elaborate, but necessary, dissection of the integuments and fascia from the muscles might have essentially indisposed the divided parts to union by adhesion.

Benjamin Cockerell, ætat. seventeen, underwent, on the 5th of March, amputation of the left thigh, for disease of the knee. I pursued the following method: With my left hand I took hold of the integuments and muscles immediately above the knee. My assistant, Mr. Tuson, in like manner grasped the flesh higher up, beyond the line which I intended the incision should take. The flesh of the thigh was then drawn to the inside, so that the line of the integument, which is naturally placed above the axis of the femur, now covered its inner margin. A long and pointed two-edged knife was then passed through the thigh, on the inside of the bone, and carried obliquely downwards and outwards, so as to cut its way out. The same steps were repeated on the inside, and the second lateral flap thus made. The bone being divided, the flaps came together perfectly. Union took place by adhesion.

This operation in many respects was satisfactory. The division of the soft parts was more rapidly executed in this manner than by circular incisions; and I thought that the pain suffered was actually less, through the parts being divided *from within outwards.* The healing of the stump was as rapid as possible, as the whole of the interior united by adhesion. This must be attributed partly to the simpleness of the wound, which is unattended by such separation and dissection of parts as takes place in the method by circular incisions; partly to the exact apposition into which the divided surfaces are brought in the flap operation, leaving no intervals or vacuities, which first fill with blood, and then suppurate.

The only point which did not perfectly answer my expectation was, that the bone, though well covered, pointed directly against the cicatrix. Nevertheless, I contrived, before the stump had completely cicatrized, to obviate this inconvenience in a great degree by rolling the thigh from within outwards, so as to twist the integuments and flesh in such a manner that the greater part of the end of the bone rested against the inner flap.

It occurred to me, on reflecting upon this case, that, by modifying the flap operation,—by making, for example, the flaps transverse, instead of lateral,—the inconvenience I have last adverted to might be possibly obviated. An opportunity offered of putting this idea in practice.

Hannah Allen underwent amputation of the thigh on the 29th of March. I employed the method of transverse flaps. I first made the posterior flap, having pierced the limb behind the bone with a long catline, which cut itself out. I then in the same manner made the second flap in front of the bone; giving it, however, greater length than the posterior flap. The long anterior flap came well before, and completely covered the bone. The stump united by adhesion.

The result of this case was highly satisfactory, as far as again showing the superior rapidity with which a stump will heal after the flap operation; and in the specific point which I had in view I was not disappointed. But an unexpected evil has shown itself, which will make me in future adopt in preference the operation by lateral flaps. The patient experiences difficulty and pain in fully extending the thigh. This is perhaps accidental; but I am disposed to attribute it in part to the rectus having been left nearly entire in the anterior flap, and having been brought down before the bone, behind which is the line of union with the posterior flap. I suppose that the tone of the rectus is constantly tending to bring the hip-joint to the state of flexion. But, as the flesh of the extremity of a stump always degenerates, I entertain hopes that in this instance the inconvenience which I have described will be temporary only, and will eventually disappear.

The great advantages which attend the flap operation are, expedition in its performance, and subsequent rapidity of union, and recovery. It has this minor advantage besides: the bone can be sawn through with more certainty at the height required than in the circular method. However, upon this point there is some uncertainty even in the flap operation; as the tendency to retract in the muscles and integuments varies so remarkably in different cases. The only law which I have observed upon this subject is, that in strong and healthy limbs the integument retracts to a great extent, the muscles hardly at all; while in extenuated limbs exactly the reverse happens.

19, *George street, Hanover square;*
*June* 3, 1834.

